# Promising Adjunct Medicines in the Protocol of COVID-19 Clinical Trial

**DOI:** 10.34172/apb.2022.067

**Published:** 2021-10-06

**Authors:** Mona Mahmoud AbouSamra, Soheir Said Mansy

**Affiliations:** ^1^Pharmaceutical Technology Department, Pharmaceutical and Drug Industries Research Division, National Research Centre, Cairo, 12622 Egypt.; ^2^Electron Microscopy Research Department, Theodor Bilharz Research Institute, Cairo, Egypt.

**Keywords:** COVID-19, Drugs, Protocol

## Abstract

Severe acute respiratory syndrome coronavirus 2 (SARS-CoV-2) is the causative organism of COVID-19. Since this disease is considered new and does not have an approved curative protocol, many researchers have tackled the possible options for COVID-19 prevention and therapeutic approaches. We address herein the phenomena of cytokine storm (the main cause of death) associating with the late stage of COVID19. Cytokine storm is undertaken in an attempt to provide information about its possible underlying causes, and to clarify some points that can be of value in guiding treatment practices for a clinical trial.

## Introduction

A severe acute respiratory pandemic syndrome known as COVID-19 caused by severe acute respiratory syndrome coronavirus 2 (SARS-CoV-2) emerged in the world, and attacking millions of people causing several deaths in 216 countries World Health Organization (WHO). Treatment modalities currently applied in the world remain under evaluation and are generally built on viral destruction and decreasing viral load.^1^

Cytokine storm is the complication of severe COVID19. It is characterized by hyper-inflammation resulting in severe dysfunction of multiple organs. It is suspected to be the main cause of death in infected patients.^2^ It remains a challenge in front of the researchers for preventing its occurrence or treating its complications. The currently used treatment is focusing on cytokine inhibitors, corticosteroids, therapeutic plasma exchange, and convalescent plasma.^1,2^ This treatment is based on the immune system deregulation as response of dendritic cells and mononuclear macrophages to viral antigens. Pro-inflammatory cytokines such as interleukin 6 (IL-6), IL-1, and tumor necrosis factor α (TNF-α) are produced. IL-6 stimulates T-cells to activate adaptive immunity. Activated T cells also stimulate macrophage and NK cells through interferon gamma (IFN-γ) to promote virus removal.^2^

Although SARS-CoV-2 viral loads, especially plasma viremia, are associated with increased risk of mortality,^3^ elevated inflammatory markers without detectable plasma viremia were also confirmed in patients with COVID19.^3,4^ Thus, we argue that the unbalance between Renin-angiotensin-aldosterone system (RAAS) with their counteract angiotensin-converting enzyme 2** (**ACE2)/angiotensin 1-7/mas receptor formation must not be neglected as possible contributors to this dilemma. We propose that the line of treatment must focus on the protective role of ACE2 in mitigating the pathological effects of ANG II which is more or less blocked by SARS-CoV-2 binding affinity to this receptor.

## ACE2 and cytokine storm

ACE2 represents the indoor of the virus to the human cells and tissues.^5-8^ It reveals its high expression in the heart and lung.^9^ The active form of ACE2 is produced by the enzyme ADAM17 or Sheddase. The cleaved ACE2 is liberated in the serum and reacts with angiotensin II produced by ACE 1 to form angiotensin 1/7. The latter, in turn, activate the mas oncogene receptor which is a powerful antioxidant, anti-inflammatory, vasodilator by the stimulation of nitric oxide (NO) release and endothelial NO synthase activation in endothelial cells, and decrease the production of aldosterone. It counterbalances the reaction of ACE1 that produces Angiotensin II which mediates its action by angiotensin II type 1 receptor (AT1R) in the renin angiotensin aldosterone system.^10-13^

In COVID-19, the role of ACE2 in degrading angiotensin II to angiotensin 1/7 is blocked by the binding affinity of SARS-CoV-2 to this aminopeptidase virus receptor. In turn, a shift of the balance toward the dominance of the ACE/angiotensin II/AT1R system over the ACE2/angiotensin 1-7/mas receptor system occurs. The noncompeting angiotensin II accumulation occurs, resulting in heath fatal illness through AT1R activation.^14^ AT1R leads to vasoconstriction, endothelial inflammation with formation of microvascular thrombi, fibrosis, production of pulmonary fibroblast procollagen, activation of oxidative stress (reactive oxygen species),^15-17^ stimulate the tissue factor expression , platelet-derived growth factor formation and proinflammatory activation with cytokine production as IL-6, IFN-γ, TNF-α, and IL-1β which contributes to cytokine storm occurring in patients with COVID-19.^18^

 For this, we can speculate that ACE2 and other components of the renin-angiotensin aldosterone system may play a pivotal role in controlling the severity and the progress of this disease to the cytokine storm stage.

Many investigators also argue that ACE2 represents a potential target for therapeutic intervention. Although, the inhibition of ACE or AT1R may stimulate negative feedback with upregulation of ACE2 the indoor of SARS-CoV-2 to inside the host cell, this will be associated with decreasing proinflammatory cytokines and initiation of IL-10 an anti-inflammatory cytokine through the induction of Ang 1/7 and Mas.^11,19-22^ As well as, ACE2 will perform its role in inactivating other targets such as bradykinin metabolites and other vasoactive peptides which might also contribute to SARS lung disease.^8,23^

## Proposed cytokine storm treatment protocol

The two known medicine that affects this system is the angiotensin converting enzyme inhibitor (ACEI) and angiotensin receptor blockers (ARBs). They are used as antihypertensive medication. At the same time, Hypertensive Patients subjected to ACEI or ARBs treatment felt great trouble after the raised debate concerning the continuation and discontinuation of ACEI and ARBs with the dominance COVID-19 illness, due to their possible upregulate function on ACE2 expression.^15,24,25^ Ferrario et al reported a 5-fold increase in ACE2 levels with lisinopril and a 3-fold increase in ACE2 levels with losartan secondary to ACEI and ARB medication respectively.^26^ The study of Furuhashi et al reported an increase excretion of ACE2 in the urine of patients on ARB olmesartan medication most probably from the upregulation mechanism.^27^ In the same context, clinical trials are running on ARBs as a therapeutic option for patients infected by SARS-CoV-2.^28^ On the other hand, other observational studies as that of Peng et al and Alburikan & Abuelizz did not find any significant difference in the proportion of patients using an ACEI or an ARB who had critical and non-critical COVID-19.^29-31^ Notably, there is no clinical or experimental evidence supporting that ARBs and ACEIs either augment the susceptibility to SARS-CoV-2 or worsen the severity and consequences of COVID-19 at present.^14^ Many hypothesizes were also postulated based on This ACE2 upregulation secondary to the inhibition of ACE by ACEI or AT1R by ARBs favor preferential binding sites for SARS-CoV-2. A high viral load was detected in patients with poor outcomes.^32^ A new hypothesis in the treatment of COVID-19 is the introduction of ADAM 17 or sheddase as a new promising line of treatment. It is involved in the activation or inactivation of diverse cell substrates: cytokines, growth factors, and their receptors as well as adhesion molecules.^33^

Based on the above-mentioned reported data, we propose a drug regimen that can be subjected to a clinical trial to test its efficacy. This treatment is a combination of the therapeutic dose of ACEI and ARBs, plus a dose of Adam17 equal to the double amount released in a normal person and the usual dose of the antiviral replication fulfilled by Remdesivir that reported clinical benefit.^34^ This combination is associated with a direct immune-modifying agent such as anakinra.^35^ We must take into consideration double the dose of ACEI and ARBs in hypertensive patients using these two medicines as line of treatment to assure proper increase of ACE2. The hypothesis of this proposed suggestion is based on the expected outcome of using the high therapeutic dose of ACEI and ARBs with a double dose of ADAM 17. The high therapeutic dose of ACEI and ARBs will assure complete blockade of AT1R, increase in ACE2 with increased formation of angiotensin 1/7 or angiotensin 1/9 and interestingly their beneficial anti-inflammatory, vascular and anti-cytokine effects. The double dose of ADAM17 will promote cleavage of ACE2 with the hope to be not accompanied by any imbalance reaction related to ADAM functions. Increase shedding of ACE2 by cleaving the anchoring of ACE2 to the cell membrane by Adam17 will decrease intact ACE2 and resulting in an increase in the levels of soluble ACE2. In consequence, shedding large amounts of ACE2 can capture SARS CoV2 kept in solution prior to it reaching cells^36^ and knocking down SARS-CoV2 cell penetration. At the same time, the cleaved ACE2 will react with angiotensin II to engendering angiotensin 1/7 which has an anti-inflammatory and vasodilator effect.^30^

## Conclusion

 It is clear that no single medication alone will be effective against these multifactorial mechanisms involved in the agonist and antagonist of the factors related to the accentuation or attenuation of the COVID-19 cytokine storm. No doubt a successful drug regimen applied as a treatment for COVID-19 must respect the normal balance between the involved factors in this dilemma and it is time to value the personalized medicine.

## Ethical Issues

 Not applicable.

## Conflict of Interest

 None.
